# Supplementary material for: DLG2–DLG4 Expression Is Associated with Improved Survival and a Synaptic Gene Signature in Lower-Grade Glioma
Source: Cancers (Basel). 2026 May 20;18(10):1646. doi: 10.3390/cancers18101646 (PMC13204321; doi:10.3390/cancers18101646)
Supplement: Supplementary file 1 [file cancers-18-01646-s001.zip › cancers-4292245-supplementary.pdf]

Supplementary Information for

DLG2–DLG4 Expression is Associated with Improved Survival and a Synaptic Gene Signature in Lower-Grade Glioma

Felipe Gaia, Henrique Ritter Dal Pizzol, Osvaldo Malafaia, Rafael Roesler and Gustavo R. Isolan

Supplementary Table S1. Panel of genes chosen as representative of an excitatory synapse and synaptic plasticity signature.

GENE	PROTEIN	SYNAPTIC FUNCTIONS	REFERENCES
ARC	Activity-regulated cytoskeleton-associated protein	AMPAR endocytosis, synaptic scaling, plasticity, long-term memory	[13,14]
CAMK2A	CaMKII alpha	LTP induction, AMPAR phosphorylation	[15,16]
CAMK2B	CaMKII beta	Actin binding, spine structural plasticity, LTP	[17,18]
DLG2	PSD-93 (SAP-93)	Scaffolds excitatory receptors at synapses	[5,6,19,20]
DLG3	SAP-102	Regulates NMDA receptor trafficking, maturation	[4,20,21]
DLG4	PSD-95	Anchors receptors, drives synaptic plasticity	[8,20,22]
DLGAP1	SAPAP1 (GKAP)	PSD scaffold linking PSD95–SHANK	[23,24]
EGR1	Early growth response protein 1	Activity-dependent transcription, plasticity	[25,26]
FOS	c-Fos	Immediate early gene, activity transcription	[27]
FOSB	FosB proto-oncogene	Sustained activity-dependent transcription factor	[28,29]
GRIA1	GluA1 (GluR1)	AMPAR subunit, synaptic strengthening	[30,31]
GRIA2	GluA2 (GluR2)	AMPAR Ca^{2+} impermeability, channel regulation	[32,33]
GRIA3	GluA3 (GluR3)	AMPAR subunit, basal transmission	[34,35]
GRIA4	GluA4 (GluR4)	AMPAR subunit, fast excitatory transmission	[34,36]
GRIN1	GluN1	Essential NMDAR subunit, channel function	[37,38]
GRIN2A	GluN2A	NMDAR subunit, synaptic maturation	[39,40]
GRIN2B	GluN2B	NMDAR subunit, plasticity signaling	[39,41]
HOMER1	Homer1	Links mGluRs to PSD scaffold	[41,42]
HOMER2	Homer2	Scaffold for mGluR signaling complexes	[43,44]
MAP2	Microtubule-associated protein 2	Dendritic stability, microtubule organization	[45,46]
NEFL	Neurofilament light polypeptide	Axonal structure, cytoskeletal support	[47,48]
NEFM	Neurofilament medium chain	Axonal caliber, structural integrity	[49]
NPAS4	Neuronal PAS domain protein 4	Activity-dependent synaptic gene regulation	[50,51]

PIK3R5	PI3K regulatory subunit gamma	PI3K signaling, synaptic plasticity	[52,53]
PPP3CA	Calcineurin A alpha	Activity-dependent phosphatase, LTD signaling	[54,55]
PRKCG	PKC gamma	Modulates synaptic signaling, plasticity	[56,57]
RASGRF1	RasGRF1	Ras activation, synaptic plasticity signaling	[58,59]
RBFOX3	NeuN	Neuronal splicing factor, identity marker	[60,61]
SATB2	SATB2	Cortical neuron identity, neuronal development, gene regulation	[62,63]
SHANK1	Shank1	PSD scaffold, spine structure	[64,65]
SHANK2	Shank2	PSD scaffold, receptor organization	[65,66]
SHANK3	Shank3	PSD scaffold, synaptic stability	[65,67]
SLC17A6	VGLUT2	Vesicular glutamate transporter	[68,69]
SLC17A7	VGLUT1	Vesicular glutamate transporter	[70,71]
SYNGAP1	SynGAP	Ras regulation, AMPAR trafficking	[72,73]
TBR1	T-box brain protein 1	Cortical neuron identity, glutamatergic differentiation	[74]

Supplementary Table S2. Cut-off values for high and low gene expression levels used for survival analyses.

**Dataset**	**Gene**	**Cutpoint**	**Low expression (*n*)**	**High expression (*n*)**
TCGA-LGG	*DLG2*	0.1234	274	216
TCGA-LGG	*DLG3*	-0.2252	205	285
TCGA-LGG	*DLG4*	-0.5811	148	342
CGGA LGG (merged 325+693)	*DLG2*	0.645	299	108
CGGA LGG (merged 325+693)	*DLG3*	-0.2327	165	242
CGGA LGG (merged 325+693)	*DLG4*	-0.6859	90	317

All expression values were standardized using gene-wise z-score transformation.

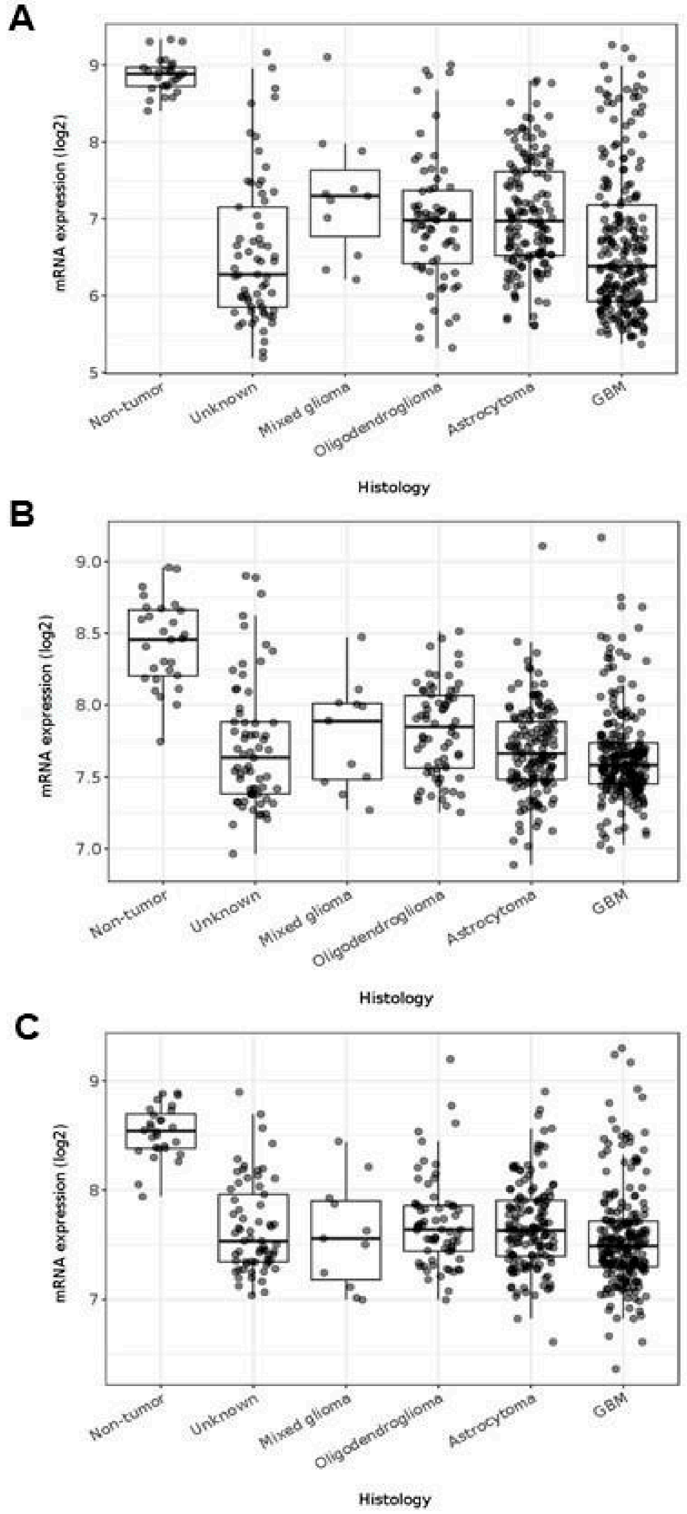

Supplementary Figure S1. Gene expression levels of (**A**) *DLG2*, (**B**) *DLG3*, and (**C**) *DLG4* in glioma types and non-tumor brain tissue. Data were obtained from the Rembrandt dataset [15] was analyzed using the GlioVis - Data Visualization Tools for Brain Tumor Datasets (https://gliovis.bioinfo.cnio.es/) platform [81].

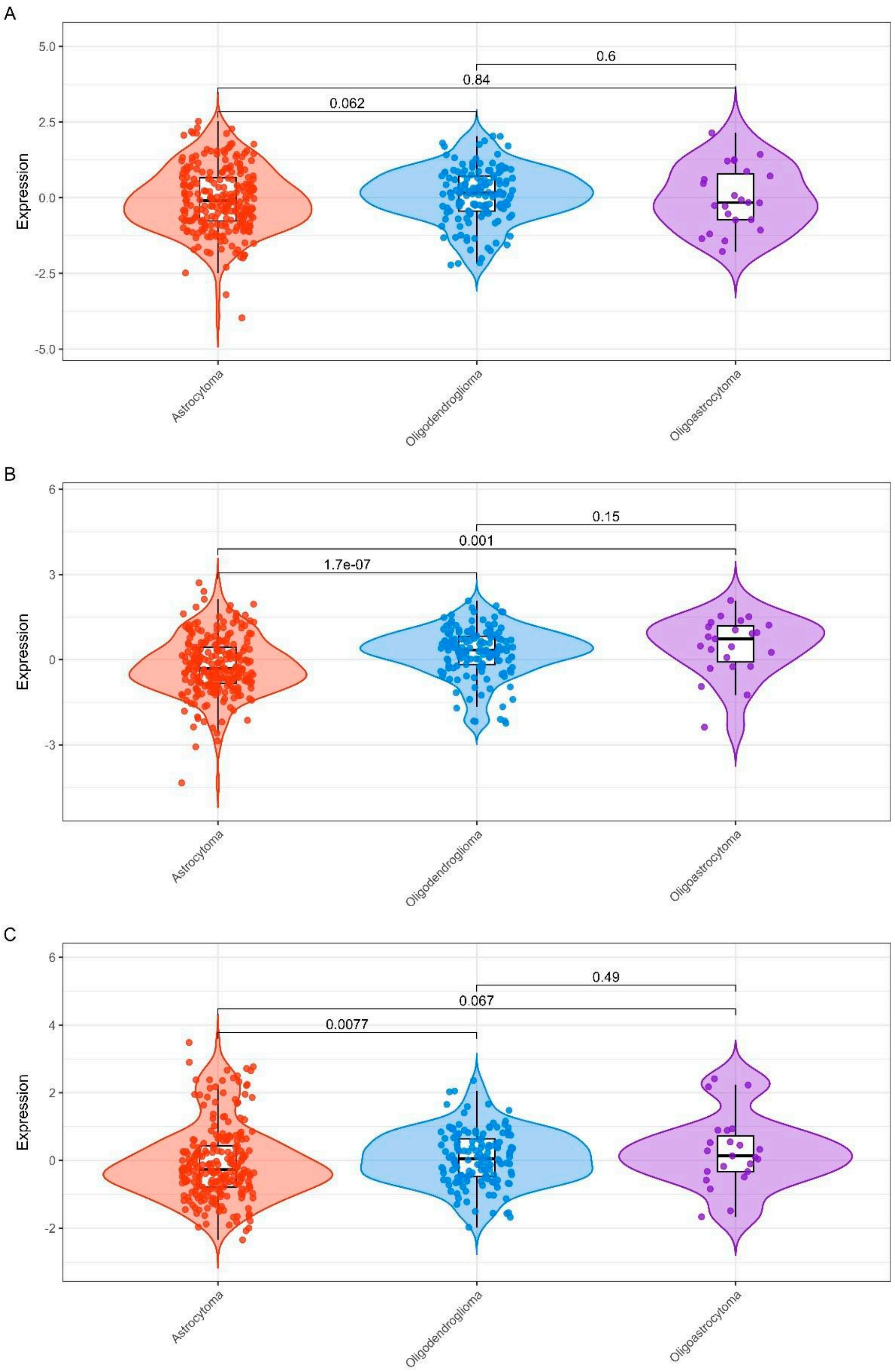

Supplementary Figure S2. Gene expression levels of (**A**) *DLG2*, (**B**) *DLG3*, and (**C**) *DLG4* in CGGA LGG tumors classified into histological types. Astrocytoma, $n = 239$; oligodendroglioma, $n = 145$; oligoastrocytoma, $n = 23$; p values are indicated in the panels.

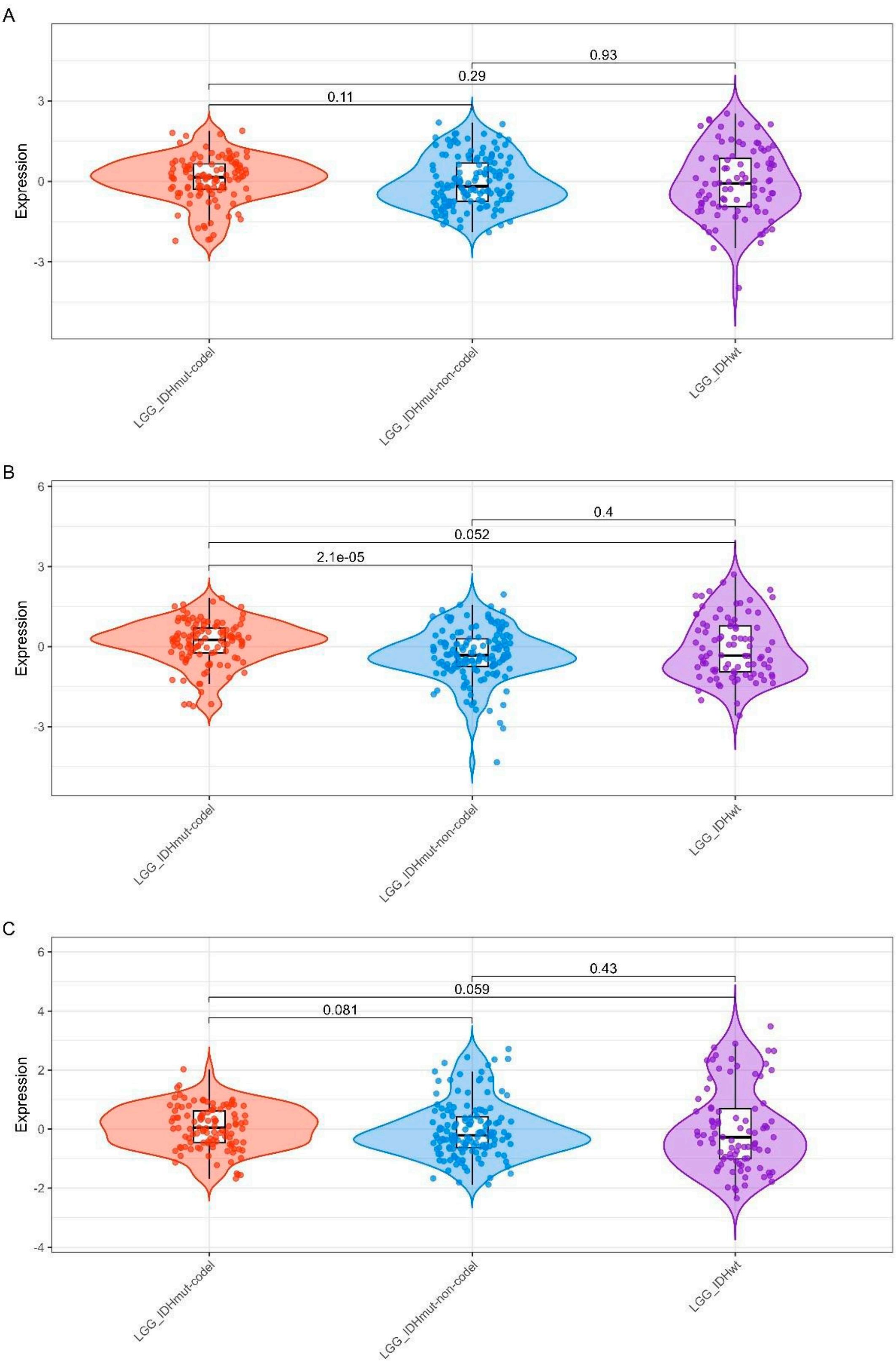

Supplementary Figure S3. Gene expression levels of (**A**) *DLG2*, (**B**) *DLG3*, and (**C**) *DLG4* in CGGA LGG tumors classified into molecular subtypes. LGG-IDH-mut-codel, n = 113; LGG-IDH- mut-non-codel, n = 142; LGG-IDH-wt, n = 88; p values are indicated in the panels.

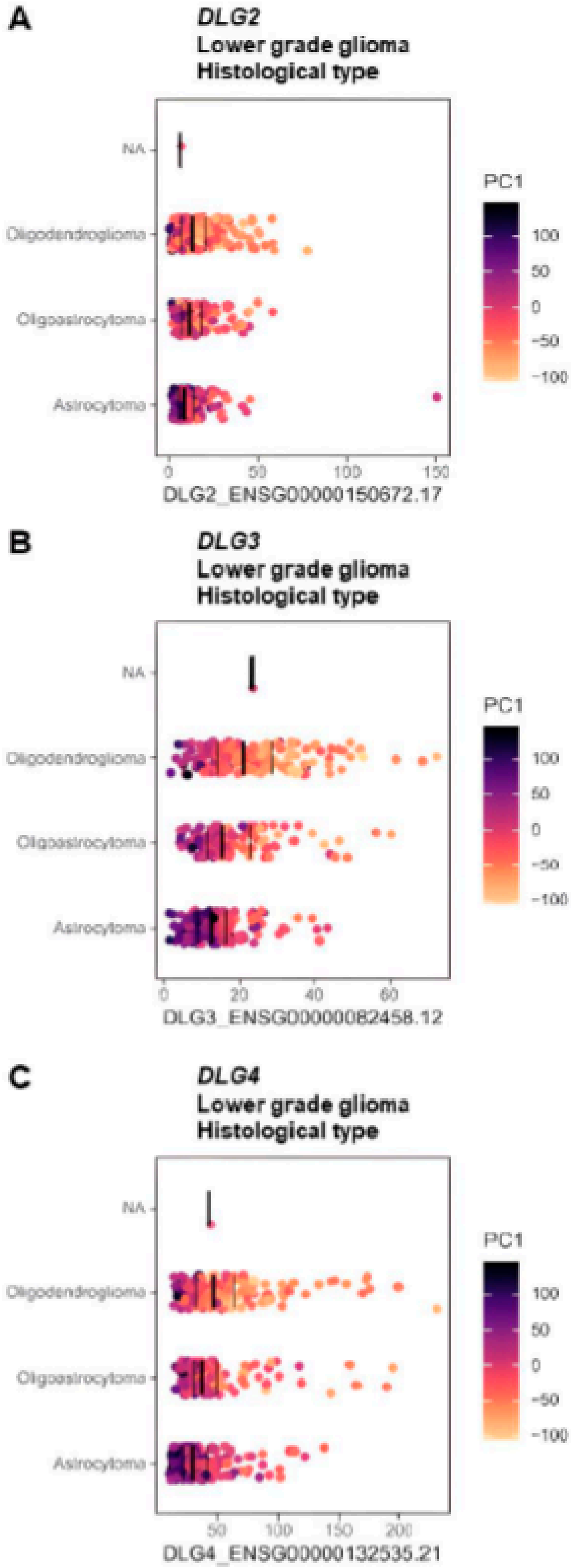
A
DLG2
Lower grade glioma
Histological type
NA
Oligodendroglioma
Oligoastrocytoma
Astrocytoma
PC1
100
50
0
-50
-100
0
50
100
150
DLG2_ENSG00000150672.17
B
DLG3
Lower grade glioma
Histological type
NA
Oligodendroglioma
Oligoastrocytoma
Astrocytoma
PC1
100
50
0
-50
-100
0
20
40
60
DLG3_ENSG00000082458.12
C
DLG4
Lower grade glioma
Histological type
NA
Oligodendroglioma
Oligoastrocytoma
Astrocytoma
PC1
100
50
0
-50
-100
50
100
150
200
DLG4_ENSG00000132535.21

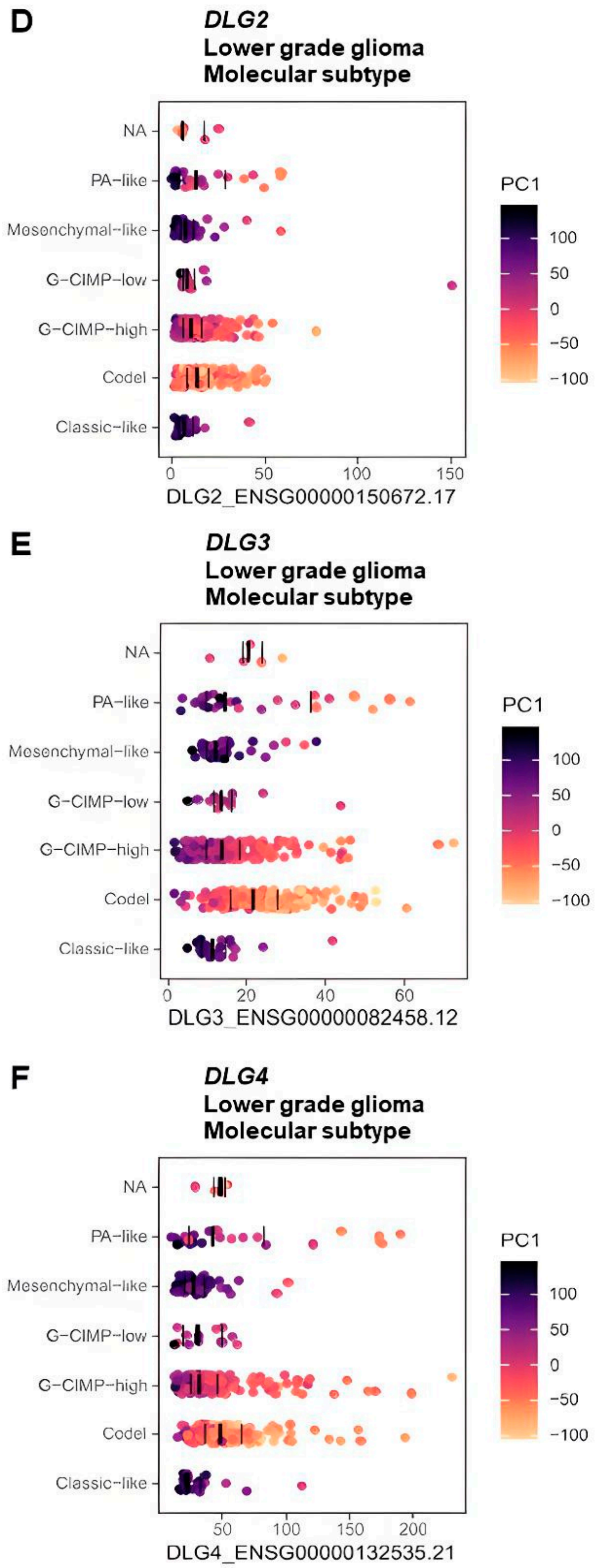

Supplementary Figure S4. Expression levels of *DLG2*, *DLG3*, and *DLG4* in relation to PC1 in TCGA LGG tumors. (**A**) *DLG2*, histological type; (**B**) *DLG3*, histological type; (**C**) *DLG4*, histological type; (**D**) *DLG2*, molecular subtype; (**E**) *DLG3*, molecular subtype; (**F**) *DLG4*, molecular subtype. For this analysis, molecular subtypes were classified as PA-like, mesenchymal-like, G-CIMP-high, G-CIMP-low, codel, classic-like, or NA [79,82].

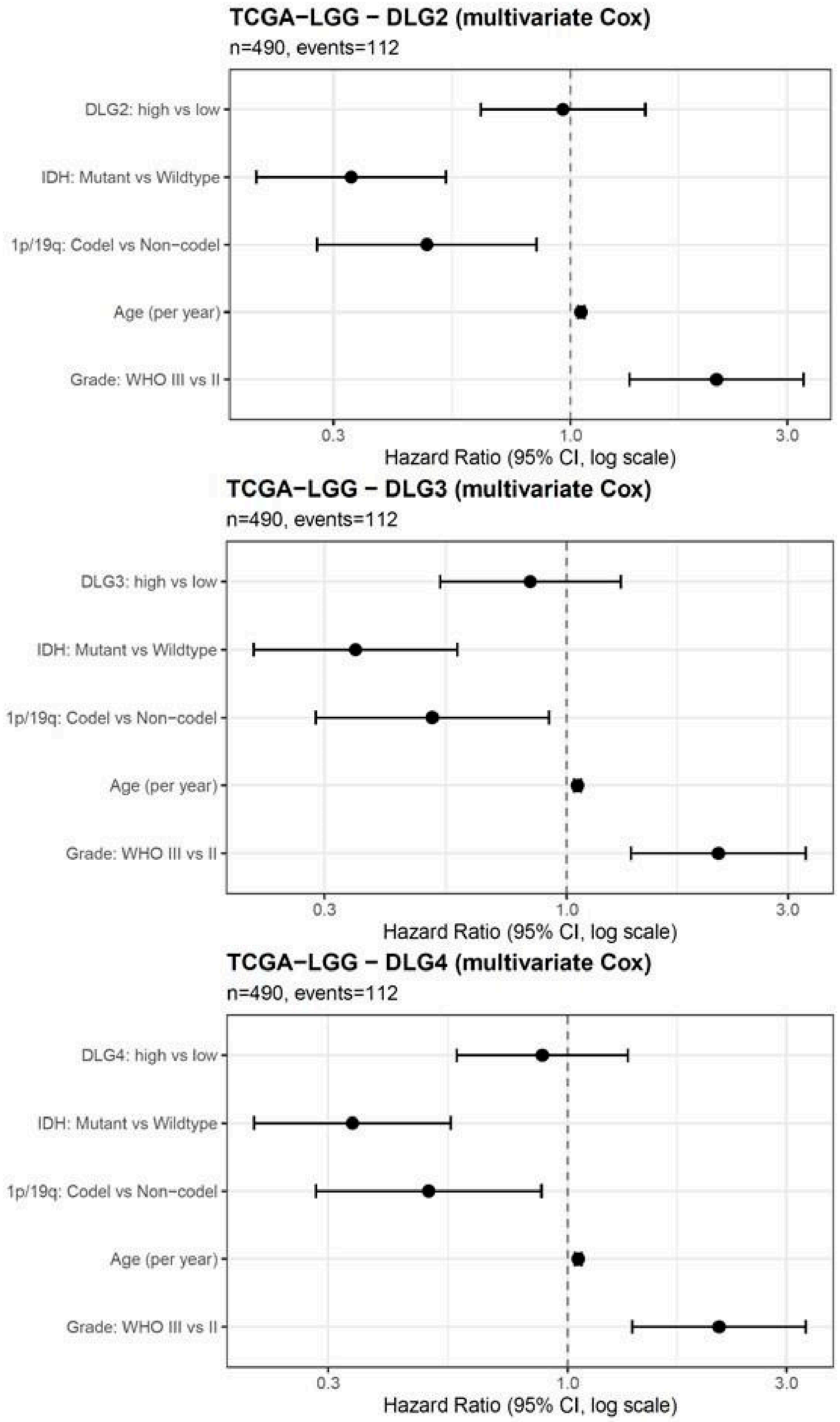

Supplementary Figure S5. Forest plots showing multivariable Cox proportional hazards analyses for OS in TCGA LLG tumors. Models included *DLG* gene expression status together with established clinicomolecular prognostic variables, namely IDH mutation status, 1p/19q codeletion status, age, and tumor grade. Hazard ratios (HRs) and 95% confidence intervals are shown. HR < 1 indicates favorable prognostic association, whereas HR > 1 indicates increased risk of death.

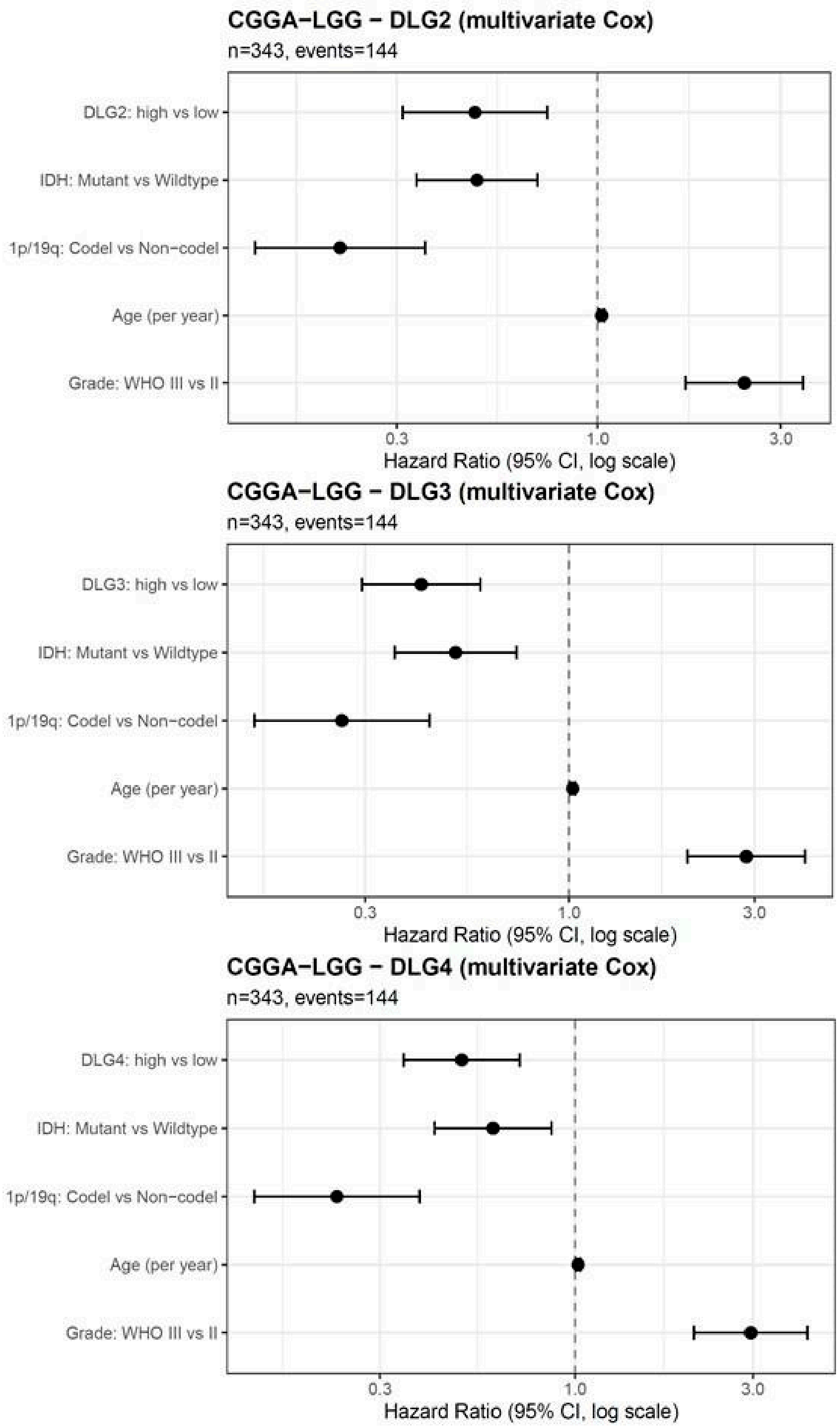

Supplementary Figure S6. Forest plots showing multivariable Cox proportional hazards analyses for OS in CGGA LLG tumors. Models included *DLG* gene expression status together with established clinicomolecular prognostic variables, namely IDH mutation status, 1p/19q codeletion status, age, and tumor grade. Hazard ratios (HRs) and 95% confidence intervals are shown. HR < 1 indicates favorable prognostic association, whereas HR > 1 indicates increased risk of death.

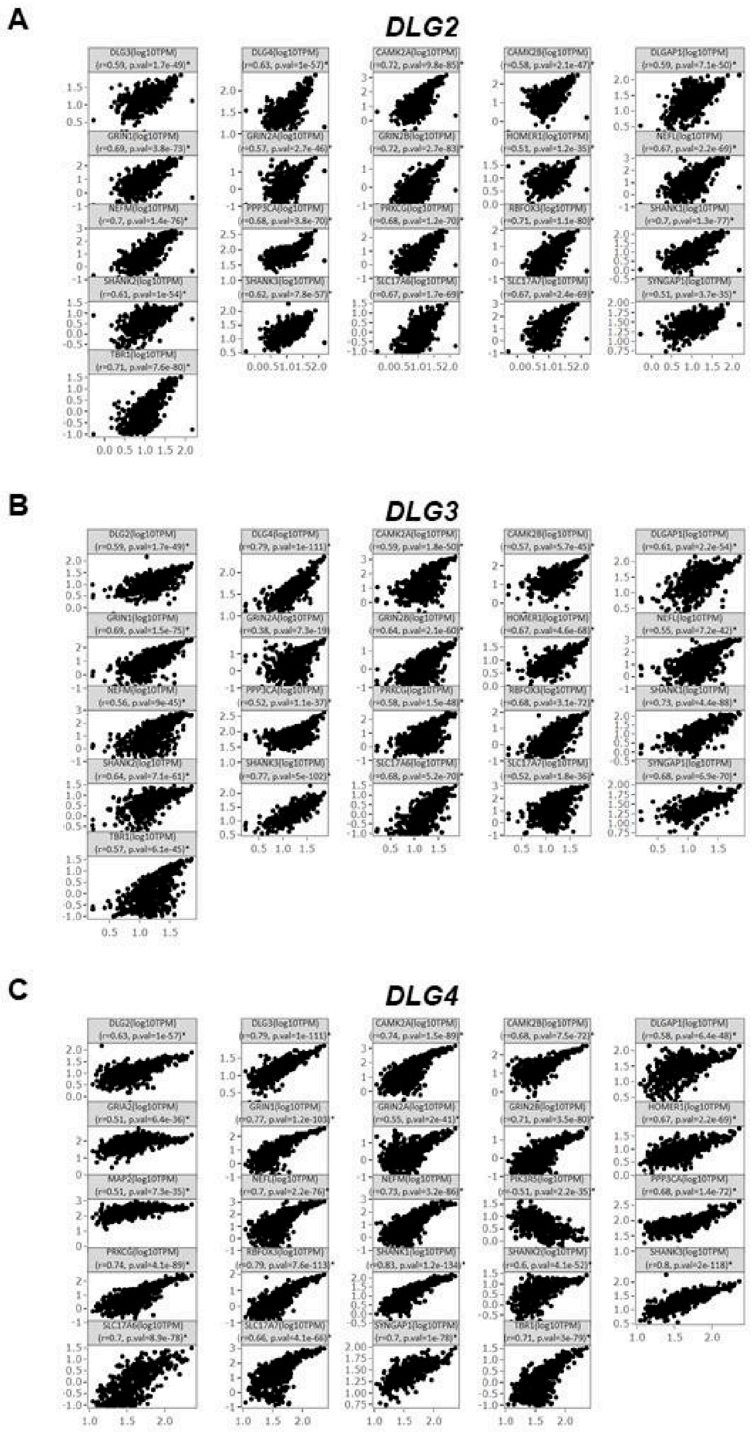

Supplementary Figure S7. Correlations found between expression of (**A**) *DLG2*, (**B**) *DLG3*, and (**C**) *DLG4* and individual genes from the synaptic dataset in TCGA LGG tumors; Pearson's r and *p* values are indicated in the panels.
